# Investigating developmental characteristics of biopsied blastocysts stratified by mitochondrial copy numbers using time-lapse monitoring

**DOI:** 10.1186/s12958-024-01262-2

**Published:** 2024-07-30

**Authors:** Chun-I Lee, Ching-Ya Su, Hsiu-Hui Chen, Chun-Chia Huang, En-Hui Cheng, Tsung-Hsien Lee, Pin-Yao Lin, Tzu-Ning Yu, Chung-I Chen, Ming-Jer Chen, Maw-Sheng Lee, Chien-Hong Chen

**Affiliations:** 1 Division of Infertility, Lee Women’s Hospital, Taichung, Taiwan; 2 Department of Obstetrics and Gynecology, Chung Shan Medical University Hospital, Taichung, Taiwan; 3 Institute of Medicine, Chung Shan Medical University, Taichung, Taiwan; 4 Department of Post-Baccalaureate Medicine, National Chung Hsing University, Taichung, Taiwan; 5 Department of Obstetrics and Gynecology and Women’s Health, Taichung Veterans General Hospital, Taichung, Taiwan; 6 School of Medicine, National Yang-Ming Chiao Tung University, Taipei, Taiwan

**Keywords:** Mitoscore, Euploidy, Morphokinetics, Time-lapse monitoring, Preimplantation genetic testing for aneuploidy

## Abstract

**Background:**

For in vitro fertilization (IVF), mitochondrial DNA (mtDNA) levels in the trophectodermal (TE) cells of biopsied blastocysts have been suggested to be associated with the cells’ developmental potential. However, scholars have reached differing opinions regarding the use of mtDNA levels as a reliable biomarker for predicting IVF outcomes. Therefore, this study aims to assess the association of mitochondrial copy number measured by mitoscore associated with embryonic developmental characteristics and ploidy.

**Methods:**

This retrospective study analyzed the developmental characteristics of embryos and mtDNA levels in biopsied trophectodermal cells. The analysis was carried out using time-lapse monitoring and next-generation sequencing from September 2021 to September 2022. Five hundred and fifteen blastocysts were biopsied from 88 patients undergoing IVF who met the inclusion criteria. Embryonic morphokinetics and morphology were evaluated at 118 h after insemination using all recorded images. Blastocysts with appropriate morphology on day 5 or 6 underwent TE biopsy and preimplantation genetic testing for aneuploidy (PGT-A). Statistical analysis involved generalized estimating equations, Pearson’s chi-squared test, Fisher’s exact test, and Kruskal–Wallis test, with a significance level set at *P* < 0.05.

**Results:**

To examine differences in embryonic characteristics between blastocysts with low versus high mitoscores, the blastocysts were divided into quartiles based on their mitoscore. Regarding morphokinetic characteristics, no significant differences in most developmental kinetics and observed cleavage dysmorphisms were discovered. However, blastocysts in mitoscore group 1 had a longer time for reaching 3-cell stage after tPNf (t3; median: 14.4 h) than did those in mitoscore group 2 (median: 13.8 h) and a longer second cell cycle (CC2; median: 11.7 h) than did blastocysts in mitoscore groups 2 (median: 11.3 h) and 4 (median: 11.4 h; *P* < 0.05). Moreover, blastocysts in mitoscore group 4 had a lower euploid rate (22.6%) and a higher aneuploid rate (59.1%) than did those in the other mitoscore groups (39.6–49.3% and 30.3–43.2%; *P* < 0.05). The rate of whole-chromosomal alterations in mitoscore group 4 (63.4%) was higher than that in mitoscore groups 1 (47.3%) and 2 (40.1%; *P* < 0.05). A multivariate logistic regression model was used to analyze associations between the mitoscore and euploidy of elective blastocysts. After accounting for factors that could potentially affect the outcome, the mitoscore still exhibited a negative association with the likelihood of euploidy (adjusted OR = 0.581, 95% CI: 0.396–0.854; *P* = 0.006).

**Conclusions:**

Blastocysts with varying levels of mitochondrial DNA, identified through biopsies, displayed similar characteristics in their early preimplantation development as observed through time-lapse imaging. However, the mitochondrial DNA level determined by the mitoscore can be used as a standalone predictor of euploidy.

**Supplementary Information:**

The online version contains supplementary material available at 10.1186/s12958-024-01262-2.

## Introduction

Mitochondria are the energy factories of cells, separated from the cytoplasm by a double membrane. They synthesize adenosine triphosphate (ATP), the essential energy currency for major cellular processes. A fully grown human oocyte is estimated to contain approximately 100,000 mitochondria. Prior to fertilization of an oocyte, the oocyte’s mitochondria are spherical with few cristae and do not actively engage in transcription or energy production. This quiescent state helps minimize the number of mitochondrial DNA (mtDNA) mutations that can be transmitted to an embryo [1]. After fertilization of the oocyte, its mitochondria undergo structural changes that, by the time of blastocyst formation, result in their resemblance to the mitochondria in somatic cells [2].

The quiet embryo hypothesis posits that embryos have evolved to minimize their metabolic activity, enabling them to survive and develop even under relatively unfavorable conditions [3]. However, recent studies have demonstrated that human embryos under stress, such as stress due to poor nutrition or environmental factors, tend to exhibit higher levels of mtDNA than do embryos not under stress. This correlation indicates that a high mtDNA level may act as a protective response to ensure embryonic cell survival during stress, potentially serving as a compensatory mechanism to provide embryos with additional chemical energy to overcome adverse conditions. In terms of clinical significance, studies have indicated a substantially lower pregnancy achievement rate with embryos that have mtDNA levels above the normal range [4, 5]. This finding indicates the importance of maintaining optimal mtDNA levels during embryo development and selection processes in assisted reproductive technologies [6].

Yu et al. (2010) revealed that mitochondria are redirected toward spindles and microtubule organization centers during oocyte maturation. This movement and distribution of mitochondria within the oocyte can affect the energy requirements for spindle formation and chromosome movement [7]. This finding indicates that the quantity, distribution, and functionality of mitochondria within an oocyte may affect the successful organization of chromosomes, leading to the formation of an aneuploid oocyte [8–10]. Moreover, embryos with high-level mosaicism were discovered to result in increased incidence of mitochondrial dysfunction, leading to decreased ATP production and impaired cellular energy metabolism during embryonic divisions, potentially causing mitotic errors [11]. Recent studies have examined mtDNA levels in biopsy specimens derived from the trophectoderm (TE) cells of blastocysts. According to several studies, mtDNA can act as an independent marker of aneuploidy, irrespective of maternal age. Results have indicated that aneuploid blastocysts generally have higher mtDNA levels than do euploid blastocysts [5, 12, 13]. However, the relationship between embryonic aneuploidy and mtDNA in TE cells remains a topic of debate [14, 15].

Early mammalian embryos exhibit dynamic metabolism that supports embryonic development and the challenge of cell fate determination. Metabolic pathways are not only essential for meeting cellular energy requirements but also play crucial roles in cellular processes, such as cell proliferation and differentiation [16]. Recent advancements in technology have enabled researchers to use time-lapse (TL) imaging for evaluating the quality of embryos by observing cleavage divisions and patterns. This innovative approach has shed light on the relationship between efficient energy metabolism and the likelihood of an embryo’s successful implantation in the uterus [17]. Using TL technology, this study examined the developmental characteristics of embryos with varying mtDNA copy numbers, as assessed using the mitoscore. Furthermore, the relationship between embryonic euploidy and the mitoscore was investigated to determine whether the mtDNA copy number can serve as an indicator of embryonic ploidy.

## Materials and methods

### Study design and patient selection

This retrospective cohort study adhered to the applicable guidelines and regulations. The study protocol was approved by the Institutional Review Board of Chung Shan Medical University, and a waiver for written informed consent was granted (approval number CS1-21156). The data used in this study, collected from Lee Women’s Hospital, were from 88 women who underwent 99 cycles of preimplantation genetic testing for aneuploidy (PGT-A) between September 2021 and September 2022. Patients who underwent a donor cycle or an autologous cycle without TL cultivation were excluded from the study.

### Embryo culture and TL evaluations

Laboratory procedures and TL observations were conducted in accordance with standard protocols outlined in our previous studies [18, 19]. Oocytes were collected through controlled ovarian hyperstimulation with either the progestin-primed ovarian stimulation protocol or the gonadotropin-releasing hormone antagonist protocol (Cetrotide; Merck Serono, Geneva, Switzerland). Ultrasound-guided ovum pickup was performed 36 h after the dual trigger, which involved the administration of human chorionic gonadotropin (250 µg, ovidrel; Merck Serono, Modugno, Italy) and triptorelin (0.2 mg, decapeptyl; Ferring, Schleswig–Holstein, Germany) to induce oocyte maturation. Following fertilization of oocytes through either intracytoplasmic sperm injection or conventional insemination, the embryos were cultured in an EmbryoScope + incubator (Vitrolife, Kungsbacka, Sweden) by using a sequential culture system from SAGE Biopharma (Bedminster, NJ, USA). The embryos were cultivated in a hypoxic environment at 37 °C under 5% O_2_, 6% CO_2_, and 89% N_2_. At 118 h post insemination, individual embryos were assessed for their morphokinetics in terms of TL characteristics as well as for any cleavage dysmorphisms and blastocyst morphology. The definitions of the observed TL parameters were provided in Supplementary Table 1. This assessment was performed using the EmbryoViewer software (Vitrolife, Kungsbacka, Sweden). The developed blastocysts were assigned scores in accordance with the KIDScore D5 (version 3.2) system by following the manufacturer’s protocols (Vitrolife, Kungsbacka, Sweden). These features and scores were collected for further analysis.

### Next-generation sequencing for PGT-A

Blastocysts were selected for analysis on day 5 (D5) or day 6 (D6) on the basis of specific criteria, including a minimum diameter of 150 μm and inner cell mass (ICM)/TE grades of ≥ BC (i.e., AA, AB, BA, BB, AC, or BC) in the Gardner embryo grading system. Subsequently, micromanipulation techniques were used to isolate five to eight TE cells from each blastocyst. The isolated cells were then rinsed and placed in a polymerase chain reaction tube. This study used the Thermo high-resolution next-generation sequencing (hr-NGS) platform to determine embryonic ploidy. The ReproSeq PGS Kit from Thermo Fisher Scientific was used to extract and amplify genomic DNA and prepare libraries from the TE biopsy samples. Automatic template preparation was conducted using the Ion Chef system, and DNA sequencing was performed using an Ion 520 chip with the Ion GeneStudio S5 system. Ion Reporter software was used for data analysis and interpretation. In addition, the mtDNA copy number of individual blastocysts was measured using the Reproseq-PGS workflow and Ion Reporter software. The process included calculating the ratio of sequencing reads between mitochondrial DNA and autosomal DNA. The mitoscore in this study was determined by multiplying the resulting values by 1000. The ploidy status of a blastocyst was determined by assessing the level of mosaicism. Blastocysts with mosaicism levels of (1) ≤ 20% in biopsied cells were categorized as euploid, (2) > 20% to < 50% were categorized as exhibiting low-level mosaicism, (3) ≥ 50% to ≤ 80% were categorized as exhibiting high-level mosaicism, and (4) > 80% were categorized as aneuploid.

### Statistical analysis

Statistical analyses were performed using SPSS Statistics (version 26.0) and GraphPad Prism (version 6.0). The biopsied blastocysts were divided into four groups based on the quartile distribution of their mtDNA copy number. This classification was implemented to facilitate clinical applications and enhance the understanding of mtDNA’s role in embryonic development. Differences between these groups were evaluated using the Kruskal–Wallis test, Pearson’s chi-squared test, or Fisher’s exact test, as appropriate. The relationships between the measured variables and the probability of euploidy were analyzed using the generalized estimating equation (GEE) method, employing both univariate and multivariate logistic regression models. Confounding variables in the dataset were identified based on their significance in univariate GEE analysis. A significance level of *P* < 0.05 was used for all statistical tests.

## Results

Embryos intended for PGT-A were cultured in a TL incubator. A total of 515 expanding blastocysts were biopsied on either D5 or D6 for the evaluation of chromosomal status through hr-NGS. These blastocysts, along with their corresponding cycle and embryo characteristics, were included in the current study and are presented in Table 1.

**Table 1:** Cycle and embryonic characteristics

Variables	Values
**Total cycles**	**99**
Female age, mean ± SD (years)	37.9 ± 4.5
AMH, mean ± SD (ng/mL)	4.0 ± 3.0
BMI, mean ± SD (kg/m^2^)	22.4 ± 3.7
Male age, mean ± SD (years)	39.2 ± 4.8
Sperm quality (%)	
Normal	66 (66.7)
Abnormal	33 (33.3)
**Total biopsied blastocysts**	**515**
Ploidy status (%)	
Euploidy	206 (40.0)
Low-level mosaicism	67 (13.0)
High-level mosaicism	39 (7.6)
Aneuploidy	203 (39.4)
Types of chromosomal abnormalities (%)	
None	206 (40.0)
Segmental chromosome alterations	56 (10.9)
Whole chromosome alterations	253 (49.1)
Embryo day (%)	
Day 5	303 (58.8)
Day 6	212 (41.2)
Blastocyst expansion levels (%)*	
Level ≤ 1	55 (10.7)
Level 2	424 (82.3)
Level 3	36 (7.0)
The grading of inner cell mass (%)*	
A	35 (6.8)
B	312 (60.6)
≤ C	168 (32.6)
The grading of trophectoderm cells (%)*	
A	21 (4.1)
B	435 (84.5)
≤ C	59 (11.5)
KIDScore D5, mean ± SD	5.7 ± 1.7
Mitoscore, mean ± SD	1.1 ± 0.6

The abbreviations “AMH”, “BMI”, “D5”, and “SD” represent “anti-Mullerian hormone”, “body mass index”, “day 5”, and “standard deviation”, respectively. *Individual embryos were assessed for their blastocyst morphology at 118 h post insemination (hpi)

### The cycle and embryonic characteristics of blastocysts with stratified mitoscores

To examine differences in cycle and embryonic characteristics between blastocysts with low versus high mitoscores, the blastocysts were divided into quartiles on the basis of their mitoscores. Group 1 had an average mitoscore of 0.58 ± 0.12, ranging from 0.2 to 0.7. Group 2 had an average mitoscore of 0.89 ± 0.08, ranging from 0.8 to 1.0. Group 3 had an average mitoscore of 1.24 ± 0.12, ranging from 1.1 to 1.4. Group 4 had an average mitoscore of 2.03 ± 0.54, ranging from 1.5 to 3.8 (Table 2). The variables considered were female age, male age, anti-Müllerian hormone (AMH) level, body mass index (BMI), KIDScore D5, the rates of full blastocysts and good blastocysts on D5, developmental kinetics, and cleavage dysmorphisms. Embryo characteristics did not significantly differ between blastocysts with low versus high mitoscores (Table 2).

**Table 2:** The cycle and embryonic differences between mitoscore groups

Mitoscore	Group 1 (0.2–0.7, *n* = 131)	Group 2 (0.8–1.0, *n* = 152)	Group 3 (1.1– 1.4, *n* = 139)	Group 4 (1.5–3.8, *n* = 93)
Mitoscore, mean ± SD	0.58 ± 0.12^abc^	0.89 ± 0.08^adf^	1.24 ± 0.12^bdg^	2.03 ± 0.54^cfg^
Female age, mean ± SD (years)	36.0 ± 5.0	36.5 ± 4.0	37.2 ± 4.5	37.7 ± 4.6
Male age, mean ± SD (years)	38.8 ± 5.4	38.6 ± 4.4	38.7 ± 5.4	38.0 ± 4.4
AMH, mean ± SD (ng/mL)	5.3 ± 2.9	4.6 ± 2.9	5.4 ± 3.4	5.0 ± 4.0
BMI, mean ± SD (kg/m^2^)	22.3 ± 3.4	21.9 ± 3.2	22.1 ± 3.7	21.8 ± 3.8
Full blastocysts on D5, % (n)	92.4 (121)	89.5 (136)	84.9 (118)	91.4 (85)
Good blastocysts (≥ 2BB) on D5, % (n)	65.6 (86)	65.8 (100)	66.2 (92)	74.2 (69)
KIDScore D5, mean ± SD	5.7 ± 1.7	5.7 ± 1.7	5.6 ± 1.8	6.0 ± 1.7

The abbreviations “AMH”, “BMI”, “D5”, “SD”, “2BB”, and “n” represent “anti-Mullerian hormone”, “body mass index”, “day 5”, “standard deviation”, “blastocysts with level 2 expansion, grade B inner cell mass, and grade B trophectoderm cells”, and “number”, respectively. Significant differences between groups were indicated by the same superscripted letters a, b, c, d, e, f, and g by using the Kruskal-Wallis, Pearson’s chi-square, or Fisher’s exact tests at a level of *P* < 0.05

### TL and ploidy characteristics of blastocysts with stratified mitoscores

**Fig. 1:**
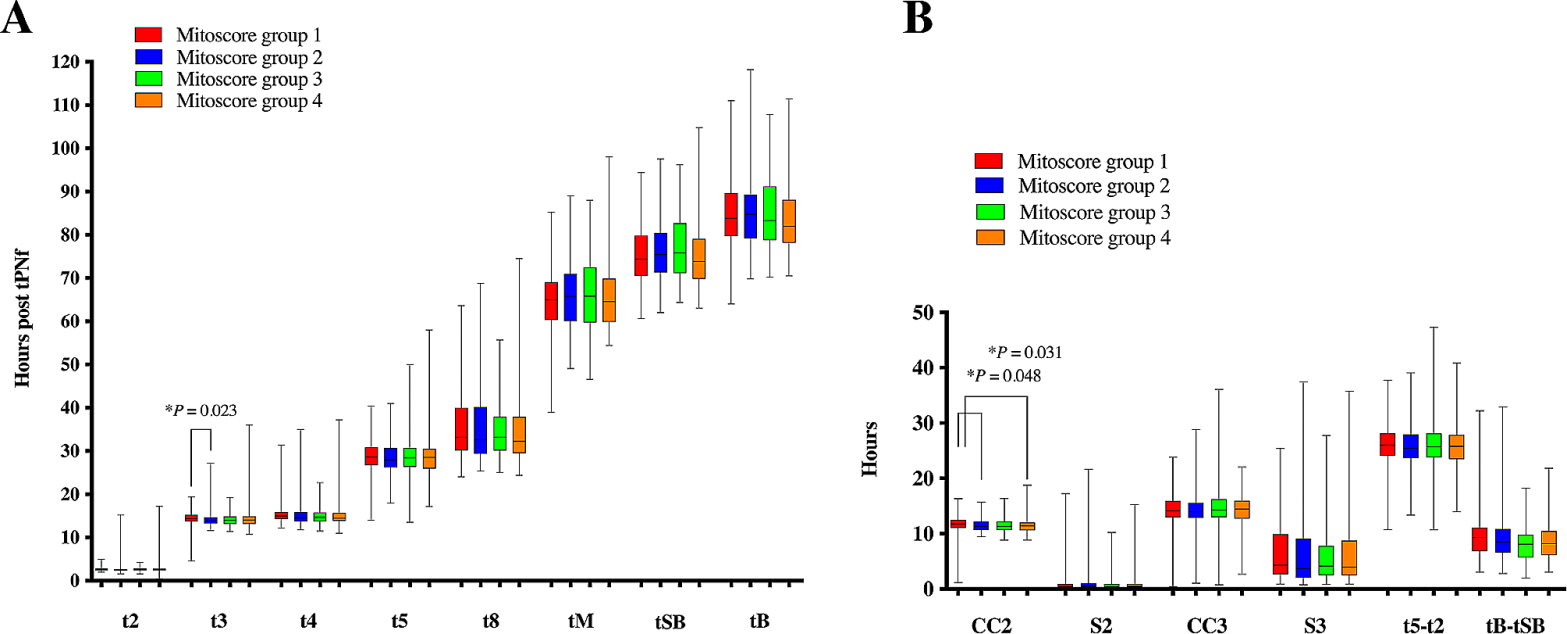
A comparison was conducted on the morphokinetic characteristics of biopsied blastocysts with different mitoscores. The boxplot shows the median, 25th/75th percentiles, and minimum and maximum timings (**A**) or time intervals (**B**) of morphokinetic parameters. Most of the observed timings and time intervals were similar between mitoscore groups. However, mitoscore group 1 showed a delayed t3 (median: 14.4 h) accompanied by a prolonged period for CC2 (median: 11.7 h). The Kruskal-Wallis test was applied for statistical analysis. Morphokinetic abbreviations are described in Supplementary Table 1

Regarding morphokinetic characteristics, no significant differences were discovered in most developmental kinetics and observed cleavage dysmorphisms (Fig. 1; Table 3). However, mitoscore group 1 had a longer time to the 3-cell stage (t3; median: 14.4 h) than did mitoscore group 2 (median: 13.8 h; *P* < 0.05). Furthermore, mitoscore group 1 had a longer second cell cycle (CC2; median: 11.7 h) than did mitoscore group 2 (median: 11.3 h) or mitoscore group 4 (median: 11.4 h; *P* < 0.05; Fig. 1).

**Table 3:** The differences in embryonic dysmorphisms between mitoscore groups

Mitoscore	Group 1 (0.2–0.7, *n* = 131)	Group 2 (0.8–1.0, *n* = 152)	Group 3 (1.1– 1.4, *n* = 139)	Group 4 (1.5–3.8, *n* = 93)
DD, %	3.1 (4)	2.0 (3)	2.9 (4)	2.2 (2)
DC, %	3.1 (4)	1.3 (2)	0.7 (1)	1.1 (1)
RC, %	3.8 (5)	4.6 (7)	3.6 (5)	3.2 (3)
ICD, %	2.3 (3)	2.0 (3)	0.7 (1)	1.1 (1)
Vacuoles, %	10.7 (14)	12.5 (19)	4.3 (6)	8.6 (8)
UD2, %	9.2 (12)	5.9 (9)	4.3 (6)	2.2 (2)
UD4, %	18.3 (24)	11.8 (18)	10.1 (14)	14.0 (13)
MN2, %	22.1 (29)	19.7 (30)	17.3 (24)	28.0 (26)
MN4, %	4.6 (6)	7.2 (11)	5.0 (7)	5.4 (5)

The abbreviation “n” represents “number”. No significant difference between groups was identified using Pearson’s chi-square or Fisher’s exact tests. Abbreviations related to dysmorphic divisions were described in the Supplementary Table 1

The ploidy status of blastocysts in different mitoscore groups was evaluated using hr-NGS (Table 4). The results demonstrated a lower euploid rate (22.6%) and a higher aneuploid rate (59.1%) in mitoscore group 4 than those in the other mitoscore groups (39.6–49.3% and 30.3–43.2%; *P* < 0.05). Moreover, the rate of whole-chromosomal alterations (63.4%) in mitoscore group 4 was higher than that in mitoscore groups 1 (47.3%) and 2 (40.1%; *P* < 0.05).

**Table 4:** The differences in ploidy status between mitoscore groups

Mitoscore	Group 1 (0.2–0.7, *n* = 131)	Group 2 (0.8–1.0, *n* = 152)	Group 3 (1.1– 1.4, *n* = 139)	Group 4 (1.5–3.8, *n* = 93)
Euploidy, % (n)	42.0 (55)^a^	49.3 (75)^b^	39.6 (55)^c^	22.6 (21)^abc^
Low-level mosaicism, % (n)	16.0 (21)	13.8 (21)	8.6 (12)	14.0 (13)
High-level mosaicism, % (n)	9.9 (13)	6.6 (10)	8.6 (12)	4.3 (4)
Aneuploidy, % (n)	32.1 (42)^a^	30.3 (46)^bd^	43.2 (60)^cd^	59.1 (55)^abc^
Segmental chromosomal alteration, % (n)	10.7 (14)	10.5 (16)	9.4 (13)	14.0 (13)
Whole chromosomal alteration, % (n)	47.3 (62)^a^	40.1 (61)^b^	51.1 (71)	63.4 (59)^ab^

The abbreviation “n” denotes “number”. Significant differences between groups were indicated by the same superscripted letters a, b, c, and d by using Pearson’s chi-square or Fisher’s exact tests at a level of *P* < 0.05

### Associations between mitoscores and euploid probability

This study analyzed associations between various variables and the probability of euploidy of elective blastocysts (Table 5). The variables included female age, AMH level, BMI, male age, sperm quality (normal or abnormal), expansion level (≤ 1, 2, or 3), TE quality (grade A, B, or ≤ C), ICM quality (grade A, B, or ≤ C), embryonic day (D5 or D6), KIDScore D5, and mitoscore. The results of the univariate logistic regression model indicated that female age (odds ratio [OR] = 0.885, 95% confidence interval [CI]: 0.835–0.938, *P* < 0.001) and mitoscore (OR = 0.575, 95% CI: 0.392–0.844, *P* = 0.005) were negatively associated with the probability of euploidy. By contrast, AMH levels (OR = 1.090, 95% CI: 1.028–1.156, *P* = 0.004) and KIDScore D5 (OR = 1.200, 95% CI: 1.078–1.336, *P* = 0.001) were positively associated with the probability of euploidy. Furthermore, the time of starting blastulation (tSB; OR = 0.954, 95% CI: 0.927–0.982, *P* < 0.005) and the time of full blastocyst (tB; OR = 0.970, 95% CI: 0.948–0.993, *P* < 0.05) were key components in morphokineitcs associated with the euploid probability (Supplementary Table 2). Variables related to high embryo quality and fast embryo development (when comparing with the reference group) were positively associated with the probability of euploidy. These variables were the expansion level (level 3 vs. level ≤ 1, OR = 3.579, 95% CI: 1.164–11.005, *P* = 0.026; level 2 vs. level ≤ 1, OR = 2.894, 95% CI: 1.428–5.866, *P* = 0.003), TE quality (grade A vs. grade ≤ C, OR = 7.833, 95% CI: 2.607–23.536, *P* < 0.001; grade B vs. grade ≤ C, OR = 2.765, 95% CI: 1.393–5.487, *P* = 0.004), ICM quality (grade B vs. grade ≤ C, OR = 1.867, 95% CI: 1.286–2.711, *P* = 0.001), and embryo biopsy day (D5 vs. D6, OR = 1.440, 95% CI: 1.015–2.041, *P* = 0.041). Furthermore, in the multivariate logistic regression model, female age, AMH levels, expansion levels, TE quality, ICM quality, embryo biopsy day, and KIDScore D5 were considered confounding variables. When these confounders were adjusted for, the mitoscore remained negatively associated with the probability of euploidy (adjusted OR = 0.581, 95% CI: 0.396–0.854; *P* = 0.006; Table 5).

**Table 5:** Logistic regression analysis for associations between euploidy and variables in this dataset

Variables	Univariate				Multivariate			
	OR	95% CI		*P*	^a^OR	95% CI		*P*
		Lower	Upper			Lower	Upper	
Female age	0.885	0.835	0.938	< 0.001	0.905	0.843	0.972	0.006
Anti-Mullerian hormone	1.090	1.028	1.156	0.004	1.018	0.952	1.088	0.603
Body mass index	0.953	0.894	1.015	0.135	–	–	–	–
Male age	0.962	0.905	1.022	0.212	–	–	–	–
Abnormal vs. normal sperm quality*	0.779	0.455	1.334	0.363	–	–	–	–
Expansion level 3 vs. ≤ 1*	3.579	1.164	11.005	0.026	1.454	0.089	23.682	0.793
Expansion level 2 vs. ≤ 1*	2.894	1.428	5.866	0.003	1.263	0.096	16.663	0.859
TE grade A vs. ≤ C*	7.833	2.607	23.536	< 0.001	2.379	0.160	35.326	0.529
TE grade B vs. ≤ C*	2.765	1.393	5.487	0.004	1.404	0.110	17.96	0.794
ICM grade A vs. ≤ C*	1.721	0.747	3.963	0.202	0.995	0.235	4.207	0.994
ICM grade B vs. ≤ C*	1.867	1.286	2.711	0.001	1.272	0.624	2.590	0.508
Embryo biopsy on D5 vs. D6*	1.440	1.015	2.041	0.041	1.058	0.658	1.701	0.816
KIDScore D5	1.200	1.078	1.336	0.001	1.054	0.805	1.379	0.701
Mitoscore	0.575	0.392	0.844	0.005	0.581	0.396	0.854	0.006

The generalized estimating equation (GEE) analysis was used for statistical analysis. The abbreviations “OR”, “^a^OR”,“CI”, “*P*”, “TE”, “ICM”, “D5”, and “D6” denoted “odds ratio”, “adjusted odds ratio”, “confidence interval”, “*P*-value”, “trophectoderm”, “inner cell mass”, “day 5”, and “day 6”, respectively. *Indication of a reference group in the GEE model

## Discussion

Quantification of mtDNA in embryos could be essential for ensuring the ATP supply necessary for proper embryogenesis and for overcoming adverse conditions. The levels of mtDNA in embryonic cells can serve as an indicator of embryonic quality [20, 21]. Traditional methods for measuring mtDNA levels, such as in situ hybridization, are laborious and difficult to interpret, leading to their limited use [14, 22]. However, recent studies have used quantitative real-time polymerase chain reaction to precisely measure mtDNA levels. This method provides a ratio of mtDNA to nuclear DNA (nDNA) that can be used to assess the quantity of mtDNA per cell [23]. Another approach involves using hr-NGS and developing an optimized algorithm to calculate mtDNA: nDNA ratios [4]. In this study, hr-NGS was employed in conjunction with the PGT-A procedure to derive a relative mtDNA copy number score, termed the mitoscore.

During the initial stages of cell division, the number of mitochondria in each blastomere decreases due to dilution without the addition of new mitochondria through biosynthesis; any adverse effect on mitochondrial numbers and function can thus negatively affect the development of an embryo before and after implantation [24]. The present study conducted on biopsied blastocysts, grouped on the basis of their mitoscore, revealed no significant differences in cycle characteristics between the groups (Table 2). Previous studies examining the relationship between the mitochondrial score and embryonic morphology have yielded conflicting results [13, 25–29]. These divergent results are attributable to several factors, including variations in the methodologies and criteria used to assess the mitochondrial score and embryonic morphology, differences in the quality and characteristics of the embryos studied, and potential confounding variables that were not considered during analysis. To more accurately understand the true relationship between the mitochondrial score and embryonic characteristics, this study employed TL monitoring and standardized assessments at a specific time point. The findings indicated that the rates of D5 full and good blastocysts were similar across the different mitoscore groups in biopsied blastocysts (Table 2).

The findings of this study demonstrated that most of the morphokinetic and dysmorphic features of the blastocysts were similar in all mitoscore groups (Fig. 1; Table 3). However, this study revealed that mitoscore group 1 exhibited an increase in t3, leading to a prolonged CC2 (Fig. 1A). The timing of the 3-cell stage and the duration of the second cell cycle (CC2) are crucial for successful implantation [30–32]. In this study, both intracytoplasmic sperm injection and conventional insemination methods were used for fertilization. To minimize variability, t3 was calculated using the time of pronuclear fading as time zero (t0), which differs from studies using the time of insemination as t0 [31]. Our findings related to t3 were not comparable to those of previous studies because of the use of different measures to assess effects. However, the definition of CC2 used in this study is identical to that used in other studies. Basile et al. (2015) performed logistic regression analysis and determined that CC2 (OR = 1.425, 95% CI = 1.025–1.981) is among the most relevant variables associated with implantation. They determined that the optimal range for CC2 is between 9 and 12 h. In our study, group 1 blastocysts (with a low mitoscore) had a longer CC2 (median: 11.73 h, 25th to 75th percentiles: 11.02 to 12.47; Fig. 1B). The percentage of CC2 in the range of 9 to 12 h was also lower in group 1 (59.5%, 78/131) than those in groups 2 (72.4%, 110/152), 3 (69.5%, 97/139), and 4 (73.1%, 68/93). Furthermore, the rate of D5 blastocysts in group 1 embryos (42.0%, 55/131) was lower than that in embryos in other groups, which ranged from 58.6 to 78.5%. Studies have reported that the mtDNA level of D5 blastocysts was higher than that of D6 blastocysts [27, 28, 33]. This could explain the lower mtDNA level in group 1 (Supplementary Fig. 1). All these results suggest that embryos in group 1, which had the lowest number of mtDNA, may have lower implantation potential. Despite some scholars suggesting a link between mtDNA copy number and embryo implantation potential, several recent studies do not support this association [12, 14, 25, 26]. Furthermore, studies have shown that mitochondrial function is altered in response to the stress induced by cryopreservation processes, such as vitrification and warming [34, 35]. A recent study by Pérez-Sánchez et al. has demonstrated that the copy number of mitochondrial DNA increases in human IVF embryos after vitrification, indicating that mitochondrial DNA can be modulated when exposed to different external factors and may not be a reliable indicator of embryonic viability [36]. Therefore, additional investigations are warranted to clarify and understand this association more comprehensively.

Several studies have investigated the relationship between mtDNA level and the ploidy status of blastocysts. These studies have consistently found that the mtDNA level is lower in the TE specimens of euploid blastocysts than in those of aneuploid ones [5, 12, 13, 33, 37–39]. Similarly, this study not only identified a higher aneuploid rate but also a higher rate of whole chromosomal alterations in the groups with elevated mtDNA levels (Table 4). Furthermore, several variables were associated with embryo ploidy in IVF cycles, including female age, blastocyst morphology, and embryo biopsy day [18, 40, 41]. Moreover, blastocyst grading based on morphokinetic parameters during preimplantation development, as evaluated by commercial algorithms such as KIDScore D5, has been observed to be potentially correlated with implantation success [42, 43]. This association may be attributable to the effect of morphokinetic parameters on the formation of aneuploid embryos, specifically concerning the dynamics of blastocyst development [18, 44, 45]. These significant confounders related to euploidy were consistently identified in this study, as indicated by the analysis of the variables listed in Table 5 and Supplementary Table 2. After these confounding factors were accounted for, the association between mitoscore and euploidy remained in the multivariate logistic regression analysis. The mtDNA level in biopsy specimens can be considered an independent factor related to ploidy status. This study determined a decreased euploid rate in mitoscore group 4 when categorizing embryos on the basis of female age (< 38 y and ≥ 38 y), blastocyst morphology (< 2BB and ≥ 2BB), embryo day (D5 and D6), and the time of starting blastocyst formation (tSB < 75.35 h and ≥ 75.35 h; Supplementary Fig. 2).

A major limitation of this study is its retrospective design, which can introduce biases, including a lack of randomization and selection bias. To confirm the findings of this study, randomized controlled trials should be conducted. To account for confounding factors, logistic regression analysis was performed. However, the inclusion of multiple blastocysts from the same couples in the dataset could have introduced bias in the estimations of regression parameters. To address this issue, the GEE method for analyzing repeated measurements was used [46]. The mitoscore was determined on the basis of biopsied blastocysts, and this approach may not be feasible for all fertilized embryos. Additionally, DNA extracted from a portion of TE cells may not accurately reflect the overall characteristics of the entire blastocyst. Moreover, this study did not consider the normalization criteria suggested by Victor et al. (2017) in the calculations. Thus, the observed difference could be attributable to the absence of the correction factor [14, 47]. Additional studies may be necessary to optimize the mitoscore calculation.

## Conclusion

This study demonstrated that blastocysts with different mtDNA levels, which were determined through biopsies, exhibited similar characteristics in their preimplantation development, as observed through TL imaging. Despite these similarities, the mtDNA level determined on the basis of the mitoscore can serve as an independent predictor of euploidy. Given the potential of mtDNA levels to predict embryonic ploidy, it is crucial to develop a non-invasive technique for measuring embryonic mtDNA levels. Such a technique would allow for the assessment of mtDNA without compromising the integrity of the embryo. When this measurement is combined with the evaluation of TL embryonic features, including morphokinetics and morphology, it would significantly enhance the accuracy of predicting embryonic ploidy.

### Electronic supplementary material

Below is the link to the electronic supplementary material.

Supplementary Material 1

Supplementary Material 2

Supplementary Material 3

## Data Availability

No datasets were generated or analysed during the current study.
