# Supplementary figures and images for: Investigating developmental characteristics of biopsied blastocysts stratified by mitochondrial copy numbers using time-lapse monitoring
Source: Reprod Biol Endocrinol. 2024 Jul 30;22:89. doi: 10.1186/s12958-024-01262-2 (PMC11290074; doi:10.1186/s12958-024-01262-2)

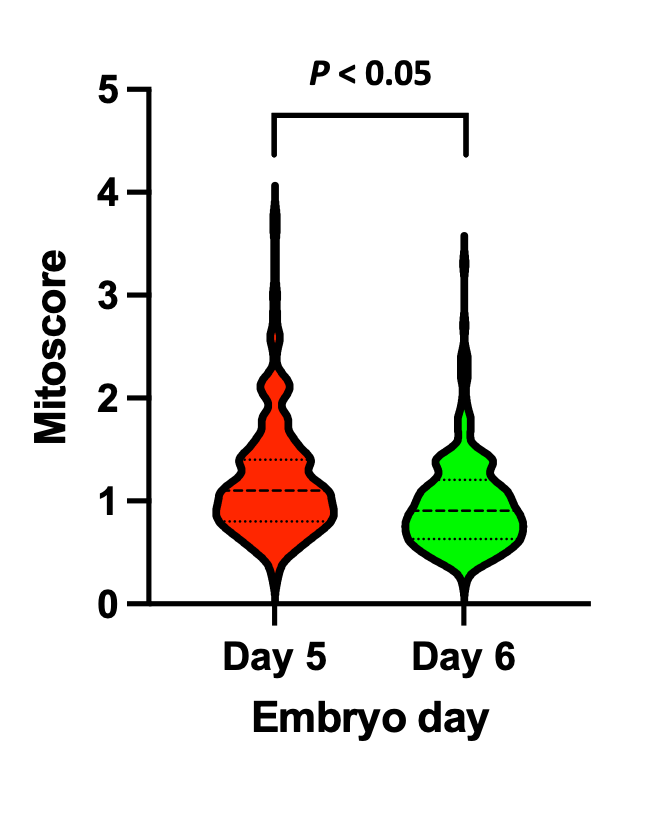

Supplement: Supplementary file 1 — Supplementary Material 1 [file 12958_2024_1262_MOESM1_ESM.tiff]

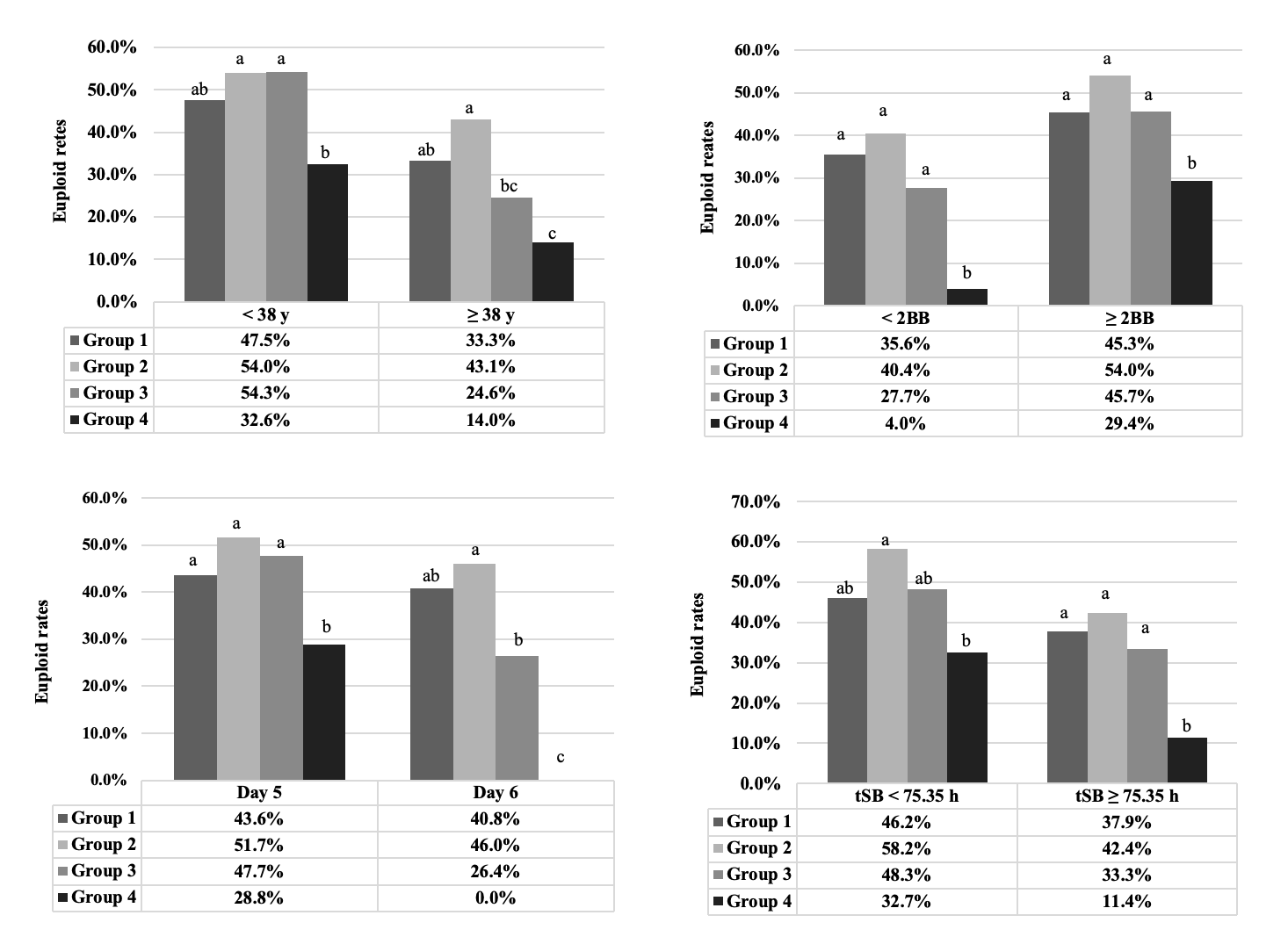

Supplement: Supplementary file 2 — Supplementary Material 2 [file 12958_2024_1262_MOESM2_ESM.tiff]
