# Supplementary material for: Investigating developmental characteristics of biopsied blastocysts stratified by mitochondrial copy numbers using time-lapse monitoring
Source: Reprod Biol Endocrinol. 2024 Jul 30;22:89. doi: 10.1186/s12958-024-01262-2 (PMC11290074; doi:10.1186/s12958-024-01262-2)
Supplement: Supplementary file 3 — Supplementary Material 3 [file 12958_2024_1262_MOESM3_ESM.docx]

Supplemental Table 1. The definition of blastocyst kinetic, morphological, and dysmorphic variables.

| Parameters | Definitions |
| --- | --- |
| tPNf | time for both pronuclei fading |
| t2 | time for embryo reaching 2-cell stage after tPNf |
| t3 | time for embryo reaching 3-cell stage after tPNf |
| t4 | time for embryo reaching 4-cell stage after tPNf |
| t5 | time for embryo reaching 5-cell stage after tPNf |
| t8 | time for embryo reaching 8-cell stage after tPNf |
| tM | time for embryo accomplishing compaction after tPNf |
| tSB | time for embryo starting blastocoel formation after tPNf |
| tB | time for blastocoel cavity starting to push zona pellucida after tPNf |
| t5-t2 | time period between t5 and t2 |
| tB-tSB | time period between tSB and tB |
| Second cell cycle (CC2) | time period between t3 and t2 |
| Synchrony in divisions for CC2 (S2) | time period between t3 and t4 |
| Third cell cycle (CC3) | time period between t3 and t5 |
| Synchrony in divisions for CC3 (S3) | time period between t5 and t8 |
| Delayed division (DD) | a single blastomere postponing division for at least one cell cycle at the first, second, or third cleavage |
| Direct cleavage (DC) | a single blastomere dividing directly or rapidly (< 5 h) from 1 cell to 3 cells at the first, second, or third cleavage |
| Reverse cleavage (RC) | abnormal cell refusion at the first, second, or third cleavage |
| Incomplete chaotic division (ICD) | a single blastomere showing a struggling division (often with formation of irregular blebbing, membrane ruffling, or pseudo-furrows) and resulting in massive fragmentation |
| Vacuole | a membrane-bound subcellular structure found in the cytoplasm of blastomeres |
| Uneven division at the 2-cell stage (UD2) | at least a 25% difference in diameter between blastomeres at the 2-cell stage |
| Uneven division at the 4-cell stage (UD4) | at least a 25% difference in diameter between the smallest and the largest blastomere at the 4-cell stage |
| Multinucleation at the 2-cell stage (MN2) | appearance of any types (e.g. single, complex) of multiple nuclei within individual blastomeres at the 2-cell stage |
| Multinucleation at the 4-cell stage (MN4) | appearance of any types (e.g. single, complex) of multiple nuclei within individual blastomeres at the 4-cell stage |
| Blastocyst expansion level ≤ 1 | non-blastocoel formation or blastocysts starting to form blastocoel |
| Blastocyst expansion level 2 | blastocoel cavity starting to push zona pellucida |
| Blastocyst expansion level 3 | blastocysts starting to herniate |
| Inner cell mass grade ≤ C | The ICM is indistinguishable or very few cells form a loosely packed cell mass with distinct boundaries. Various ICM sizes may observed in this group because of uneven cell sizes and poor compaction. The layer can be not homogenous with vacuoles, degenerated cells or independent cells. |
| Inner cell mass grade B | Several cells form the a less tightly packed cell mass. The layer can be less homogenous with few vacuoles or minor degenerations. |
| Inner cell mass grade A | Many cells form a tightly packed cell mass without distinct boundaries. The layer is homogenous without vacuoles and debris. |
| Trophectoderm cell grade ≤ C | The TE is indistinguishable or very few and larger cells often stretches over a large area. Cell cytoplasm often appears non-homogenous and vacuoles may be present. |
| Trophectoderm cell grade B | Several cells (often >20) are shown. The layer is not completely organized and the shape of the cells varies within the layer. Cell cytoplasm may appear non-homogenous and cell nuclei may be difficult to distinguish. |
| Trophectoderm cell grade A | Many flattened cells (often >40) forms a organized layer that lines the blastocoel cavity. Cell cytoplasm is homogenous and cells nuclei are often clearly visible. |

Supplementary Table 2. Univariate Logistic Regression Analysis for Associations of Euploidy with Kinetic Variables.

| Variables | Univariate | | | |
| --- | --- | --- | --- | --- |
|  | OR | 95% CI | | *P* |
|  |  | Lower | Upper |  |
| t2 | 0.887 | 0.608 | 1.293 | 0.533 |
| t3 | 0.941 | 0.830 | 1.065 | 0.334 |
| t4 | 0.989 | 0.920 | 1.064 | 0.776 |
| t5 | 0.993 | 0.946 | 1.042 | 0.779 |
| t8 | 0.985 | 0.964 | 1.007 | 0.176 |
| tM | 0.980 | 0.956 | 1.005 | 0.120 |
| tSB | 0.954 | 0.927 | 0.982 | 0.001 |
| tB | 0.970 | 0.948 | 0.993 | 0.011 |
| tB–tM | 0.974 | 0.943 | 1.006 | 0.108 |
| tB–tSB | 1.022 | 0.975 | 1.070 | 0.369 |
| CC2 (t3–t2) | 0.946 | 0.807 | 1.108 | 0.491 |
| S2 (t4–t3) | 1.037 | 0.938 | 1.146 | 0.476 |
| CC3 (t5–t3) | 1.008 | 0.949 | 1.070 | 0.804 |
| S3 (t8–t5) | 0.980 | 0.954 | 1.007 | 0.149 |
| t5–t2 | 0.998 | 0.946 | 1.053 | 0.942 |

The generalized estimating equation (GEE) analysis was used for statistical analysis. The abbreviations “OR”,“CI”, “*P*”denoted “odds ratio”, “confidence interval”, “*P*-value”, respectively. Morphokinetic abbreviations were described in the Supplementary Table 1.
